# Spatial influencing factors and protection strategies of traditional villages in ethnic areas: A case study of Guangxi

**DOI:** 10.1371/journal.pone.0336147

**Published:** 2026-04-21

**Authors:** Jianmei Tan, Jiayan Liu, Yi Zou, Xiaoshu Wei

**Affiliations:** 1 School of Management Science and Engineering, Guangxi University of Finance and Economics, Nanning, China; 2 Guangxi Laboratory for Geographic Conditions Monitoring and Urban-Rural Planning, Nanning, Guangxi, China; 3 School of Geographical Science and Planning, Nanning Normal University, Nanning, China; Hunan University, CHINA

## Abstract

The Guangxi Zhuang Autonomous Regionis an example of a concentrated settlement of ethnic minorities, characterized by a multifaceted socio-cultural environment that integrates diverse ethnic traditions. To investigate the spatial distribution patterns and driving factors of the 342 nationally recognized traditional villages in Guangxi, we adopted adopts an integrated methodological framework comprising the nearest neighbor index (NNI), imbalance index, kernel density estimation (KDE), and optimal parameters-based geographical detector (OPGD). We analyzed the differences in influencing factors between Guangxi and its surrounding regions and finally propose protection strategies for traditional villages in Guangxi. The outcomes of the research are outlined below: (1) The spatial distribution demonstrates significant clustering tendencies with pronounced regional imbalances. (2) The spatial distribution manifests a characteristic “one-primary core, three-secondary cores and dual spatial belts” pattern. (3) The spatial pattern of villages is jointly shaped by three interacting forces: natural conditions as the foundational basis, economic strength as the supporting guarantee, and socio-cultural factors as the animating core. Their coordination and mutual reinforcement jointly facilitate the distinctive development and continuity of traditional villages in ethnic minority regions. (4) This study constructs an integrated four-component conservation framework oriented toward spatially differentiated protection, reinforcement of core driving forces, optimization of the natural foundation, and activation of multidimensional synergy. The results can provide a reference for the policies and planning of the evolution of traditional settlements within ethnic minority regions.

## 1. Introduction

Traditional villages represent a fundamental pillar of China’s agricultural civilization, embodying its historical roots and cultural continuity. They are widely recognized as “living fossils” embodying rural historical, cultural, and natural heritage. The Chinese government has consistently prioritized the “preservation of traditional villages and rural distinctiveness through categorical governance.” Since 2012, China has officially designated 8,155 nationally protected traditional villages across six batches, establishing a policy framework for their conservation. Paradoxically, accelerated urbanization has intensified village hollowing out of villages. Against this backdrop, spatial configuration analysis and revitalization strategies have emerged as critical focus for traditional villages under China’s Rural Revitalization Strategy, driving interdisciplinary research at the nexus of cultural sustainability and regional development.

Known internationally as “rural settlements”, research on traditional villages originated in 1841 when a German geographer conducted systematic studies on the formation processes of human settlements. Subsequent scholars have comprehensively analyzed the relationships between settlement environments and developmental trajectories, establishing foundations for “human–environment interaction” research. Current scholarship prioritizes research on rural settlement economies [1], traditional village landscapes [2], and sustainable development [3–4].

Spatial distribution has remained a predominant topic in domestic research on traditional Chinese villages. Extant scholarship primarily operates across two analytical scales: micro-scale investigations encompass settlement-level spatial morphology, typological characteristics, and formative mechanisms; macro-scale examinations address regional structural patterns, distribution dynamics, and determinant factors in village clusters. Researchers have utilized statistical methods and GIS-based spatial analytical techniques to examine the distribution patterns and influencing factors across national [5–8] and trans-regional scales (encompassing southwest China [9–11], the Yangtze River Economic Belt [12], watershed systems [13,14], the Yangtze River Delta [15], mountain ranges [16], and provincial levels [17–21]). Research coverage now extends to all provinces with significant traditional village concentrations, with comprehensive analyses of natural, historical, and socio-cultural determinants.

Existing research indicates that the distribution of villages is shaped by a complex interplay of human and natural factors. Key environmental influences include topography, hydrology, and climatic conditions, while socio-economic variables such as economic development, urbanization, transportation infrastructure, and historical–cultural context also exert significant impact. Methodologically, prior studies have primarily integrated qualitative interpretation with quantitative techniques. Tools such as the nearest neighbor index, kernel density estimation, multi-scale geographically weighted regression model, and geographical detectors are frequently applied to examine the spatiotemporal patterns and historical dynamics [22–24]. Qualitative research methods such as local chronicles are also used to analyze the causes of spatiotemporal distribution.

While there have been substantial scholarly achievements in domestic and international research on traditional villages, there are significant opportunities for expansion in four critical dimensions: regional coverage, methodological approaches, factor analysis and protection strategies. Existing studies have primarily focused on regions in central and eastern China with deep-rooted traditional culture, as well as areas surrounding Guangxi [25–28], while relatively limited attention has been paid to ethnic minority regions such as Guangxi itself. Within the limited research concerning Guangxi’s traditional villages, the academic focus has skewed toward cultural preservation, with markedly insufficient investigation into spatial distribution patterns and underlying formation mechanisms. Research on traditional villages within the Dong ethnic settlement corridors across Hunan, Guangxi, and Guizhou remains limited, particularly with regard to studies on Guangxi and Guilin [29–32]. However, existing studies are restricted to the first five batches of nationally designated traditional villages (n = 280), omitting the more recently approved sixth batch. Consequently, they no longer provide a comprehensive representation of the spatial configuration of traditional rural settlements in Guangxi. Additionally, the existing literature has not explored the impact of factors such as the culture and population of ethnic minorities on spatial distribution. Methodologically, conventional geographical detector approaches are constrained by their reliance on manual discretization of continuous variables, introducing subjective biases that compromise detection efficacy and analytical robustness.

Guangxi is a long-settled region inhabited by the Zhuang, Yao, Miao, Dong, and other groups, and is located at the interface between China and ASEAN. What is the spatial pattern of traditional villages in this region, and how can the relative contributions of natural, economic, and socio-cultural factors to this spatial pattern be quantified? What are the underlying mechanisms through which these factors shape the spatial pattern of traditional villages? This study takes 342 national-level traditional villages in Guangxi as the research objects. It examines the spatial pattern characteristics of traditional villages in ethnic minority regions and employs OPGD to identify the major driving factors and their interactive effects. On this basis, the study analyzes the mechanisms influencing the spatial distribution of traditional villages in Guangxi and proposes targeted conservation strategies. The findings contribute to a deeper understanding of the distribution mechanisms of traditional villages in ethnic minority regions and provide evidence to support the conservation and development of traditional villages in ethnic minority areas.

## 2. Materials and methods

### 2.1. Study area profile

Geographically located in southwestern China, Guangxi features a complex and varied terrain, with mountainous and hilly landscapes constituting its predominant geographical characteristics. The region is characterized by a temperate climate, plentiful precipitation, and an extensive river system. The region is a multi-ethnic area, home to a substantial population of ethnic minority groups. Based on data from China’s seventh national population census conducted in 2020, a total of 56 ethnic groups were registered in the region, with the ethnic minority population reaching 18.808 million, accounting for 37.52%. The number of ethnic minority residents is the highest in China, with 3.1719 million more than Yunnan, which is in second place and constitutes 15.0% of the national ethnic minority population. Over the long period of interaction, the various ethnic groups have formed a residential pattern of large mixed settlements and small concentrated settlements, and together have given birth to unique ethnic cultures. By 2024, the study area had 342 villages included in the catalog of traditional Chinese villages. This number makes the region rank tenth nationwide in terms of the total count of officially designated traditional villages.

### 2.2. Data sources

The data used in this study include both basic geographic data and thematic attribute data, providing empirical support for analyzing the spatial pattern of traditional villages in Guangxi and its influencing factors. The main datasets and their sources are summarized in Table 1.

**Table 1:** Main datasets and sources.

Dataset	Year(s)	Source
Guangxi administrative boundary (vector)	2024	Tianditu National Platform for Common Geospatial Information Services (Map approval No. **GS (2024) 0650**) (https://cloudcenter.tianditu.gov.cn/administrativeDivision)
Attribute and vector data for the first–sixth batches of ancient/traditional villages in Guangxi	2012, 2013, 2014, 2016, 2019, 2023	The lists of the first–sixth batches of **national-level traditional villages** were obtained from the website of the Ministry of Housing and Urban-Rural Development (MOHURD) (accessed **25 April 2025**)(https://www.mohurd.gov.cn); geographic coordinates were retrieved using the Baidu Map coordinate-picking tool (accessed **25 April 2025**) (https://lbs.baidu.com/maptool/getpoint)
DEM raster data	2024	SRTM DEM (90 m resolution), Geospatial Data Cloud (accessed **27 April 2025**) (https://www.gscloud.cn)
Air temperature raster data	2003–2023	1 km resolution, Data Center for Resources and Environmental Sciences, Chinese Academy of Sciences (RESDC) (accessed **27 April 2025**) (https://www.resdc.cn)
Precipitation raster data	2003–2023	1 km resolution, RESDC (accessed **27 April 2025**) (https://www.resdc.cn)
Rivers and water bodies (vector)	2025	OpenStreetMap (OSM) open-data platform (accessed **27 April 2025**) (https://osmchina.org)
GDP of 14 prefecture-level cities in Guangxi	2013–2022	Guangxi Statistical Bureau: *Guangxi Statistical Yearbook* (2014–2023 editions) (accessed **27 April 2025**) (http://tjj.gxzf.gov.cn)
Road network (vector)	2025	OpenStreetMap (OSM) open-data platform (accessed **27 April 2025**) (https://osmchina.org)
Ethnic minority population	2020	Main data bulletin of the Seventh National Population Census of Guangxi (accessed **27 April 2025**) (http://tjj.gxzf.gov.cn/zt/lsgd/rkpc/t8851502.shtml)
Urbanization rate of permanent residents	2022	*2023 Statistical Bulletin on National Economic and Social Development* for each prefecture-level city (accessed **1 May 2025**) (http://tjj.gxzf.gov.cn)
Number of national-level intangible cultural heritage items	2006, 2008, 2011, 2014, 2021	China Intangible Cultural Heritage Network: lists of the first–fifth batches of national-level ICH (accessed **1 May 2025**) (https://www.ihchina.cn)
Number of autonomous region–level intangible cultural heritage items	2007, 2008, 2010, 2012, 2014, 2016, 2018, 2020, 2023	Guangxi Zhuang Autonomous Region Department of Culture and Tourism: lists of the first–ninth batches of regional-level ICH (accessed **1 May 2025**) (http://wlt.gxzf.gov.cn)

### 2.3. Research methods

#### 2.3.1 Nearest neighbor index (NNI).

The nearest neighbor index (NNI) is a quantitative method used to characterize the spatial distribution pattern of point features. By calculating the ratio of the observed nearest-neighbor distance to the expected nearest-neighbor distance under a theoretical random distribution, NNI is used to determine the type of spatial distribution exhibited by the study objects [33]. In this study, the method is used to assess the overall spatial distribution characteristics of traditional villages. The calculation is performed using the following formula:


$$R=\frac{\overset{―}{r_{i}}}{\overset{―}{r_{E}}}=\text{2}\sqrt{D}\times \overset{―}{r_{i}}$$ (1)

$$\overset{―}{r_{E}}=\frac{\text{1}}{\text{2}\sqrt{\frac{\text{n}}{A}}}=\frac{\text{1}}{\text{2}\sqrt{D}}\;$$ (2)

In the formula, $\overset{―}{r_{i}}$ and $\overset{―}{r_{E}}$ represent the actual observed and theoretical nearest neighbor distances, respectively. *D* represents the density of points. n denotes the overall count of points. *A* represents the total area of the study region. An *R* value of 1 indicates a random spatial distribution of point features. When *R* > 1, the distribution tends toward uniformity, whereas *R* < 1 signifies a clustered spatial pattern.

#### 2.3.2. Imbalance index (*S*).

The imbalance index (S) is a quantitative indicator used to measure the degree of distributional evenness of geographic elements across different regions [34]. *S* is used to assess the degree of distributional equity of traditional villages across various cities. It is calculated with the following equation:


$$S=\frac{\sum_{i=1}^{n}Y_{i}-50(n+1)}{100n-50(n+1)}$$ (3)

*n* in the formula denotes the number of prefecture-level cities. $Y_{i}$ refers to the percentage of traditional villages in each city relative to the total number in the region, and is the cumulative percentage after arranging *i* in descending order; the value of *S* ranges between 0 and 1. An *S* value of 0 indicates a perfectly balanced distribution of traditional villages across all cities, whereas an *S* value of 1 signifies complete concentration within a single city.

#### 2.3.3. Kernel density estimation (KDE).

KDE is used to characterize the spatial density of geographic features or data samples, thereby providing an intuitive visualization of whether the study objects exhibit spatial clustering or dispersion [35]. The formula used for this calculation is provided below:


$$F\left(x\right)=\frac{\text{1}}{nh}\sum_{i=1}^{n}K\left(\frac{x-x_{i}}{h}\right)$$ (4)

Within this equation, *n* refers to the total number of entities under investigation. *h* is the bandwidth, that is, the search radius, which is used to determine the local range of the analysis; $x-x_{i}$ represents the distance from point *x* to the core point $x_{i}$; $K\left(\frac{x-x_{i}}{h}\right)$ is the kernel function, which is used for smoothing. A higher kernel density value indicates a greater concentration of research objects within a given area.

#### 2.3.4. Optimal parameters-based geographical detector (OPGD).

The Geodetector is a statistical method used to detect spatial heterogeneity and to quantify the explanatory power of potential driving factors behind geographical phenomena. It can quantitatively describe the relative importance of influencing factors by constructing the statistic q value to measure the explanatory power of each factor on traditional villages. GeoDetector comprises four main modules: the factor detector, interaction detector, risk detector, and ecological detector [36].

OPGD builds upon the core functions of the four conventional GeoDetector modules, with a particular emphasis on improving the objectivity and accuracy of parameter selection. The GD package is a dedicated R package for conducting GeoDetector analyses; it relies on the core function *gdm()* to provide a one-stop workflow for optimal-parameter screening and GeoDetector-based detection. Specifically, the function *discmethod* is used to select the discretization method, and *discitv* is used to specify the candidate numbers of classes [37–38].

In this study, OPGD is applied—after parameter optimization—to analyze the drivers of spatial heterogeneity, focusing on the factor detector and the interaction detector. (1) The factor detector evaluates the explanatory power of a single factor using the q statistic q ranges from 0 to 1, with values closer to 1 indicating a stronger ability of the factor to explain spatial differentiation in the target variable [39]. (2) The interaction detector addresses the fact that, in real geographic systems, observed phenomena are often shaped by the joint effects of multiple factors. By assessing whether the joint effect exhibits enhancement, weakening, or nonlinear effects, and whether the combined explanatory power exceeds that of each factor acting alone [40], we can reveals the compound driving mechanisms underlying the spatial distribution of traditional villages. The mathematical expression for the calculation is given as follows:


$$q=1-\frac{\text{1}}{N\sigma^{\text{2}}}\sum_{\text{h}=1}^{L}N_{h}\overset{\text{2}}{\underset{h}{\sigma }}$$ (5)

where h takes the values 1, 2, …; L represents the layer of variable Yor factor X; $N_{h}$and N are the number of units (sample size) in layer h and the entire region, respectively; and $\overset{\text{2}}{\underset{\text{h}}{\sigma }}$ and $\sigma^{\text{2}}$ are the variances of *h* and *Y*, respectively. The factor’s explanatory strength lies within the interval [0, 1]. A value approaching 1 indicates a stronger capacity of the factor to explain spatial differentiation in the variable.

In the methodological framework of this study, NNI quantifies whether the overall distribution of traditional villages in Guangxi is clustered, random, or dispersed, thereby identifying the region-wide distribution pattern. The imbalance index reveals disparities in the allocation of village numbers across prefecture- and county-level units; together with NNI, it provides a complementary assessment of the overall distribution type, laying the foundation for subsequent analyses of spatial patterns and underlying mechanisms. KDE offers a visual representation of clustering patterns by transforming discrete village locations into a continuous density surface, which enables the identification of hot and cold spots and the derivation of detailed spatial distribution features. The OPGD is then employed to identify the key factors and interactive driving effects shaping the distribution of ancient/traditional villages, thereby elucidating the mechanisms through which multiple factors—such as natural conditions, socio-cultural context, and economic development—influence the observed spatial pattern. Collectively, these four methods complement one another and constitute a complete technical workflow from describing spatial patterns to uncovering influencing mechanisms.

## 3. Spatial distribution characteristics

### 3.1. Spatial distribution pattern

The NNI results show that $\overset{―}{r_{E}}=13157.08$, $\overset{―}{r_{i}}=8887.97$, R=0.67552. Z=−11.48, P=0.00.

Accordingly, the spatial distribution of traditional villages in Guangxi exhibits a pronounced clustering pattern, with only a small number of randomly distributed scattered villages appearing on the periphery of the clustering core.

### 3.2. Spatial distribution equilibrium

**Fig 1:**
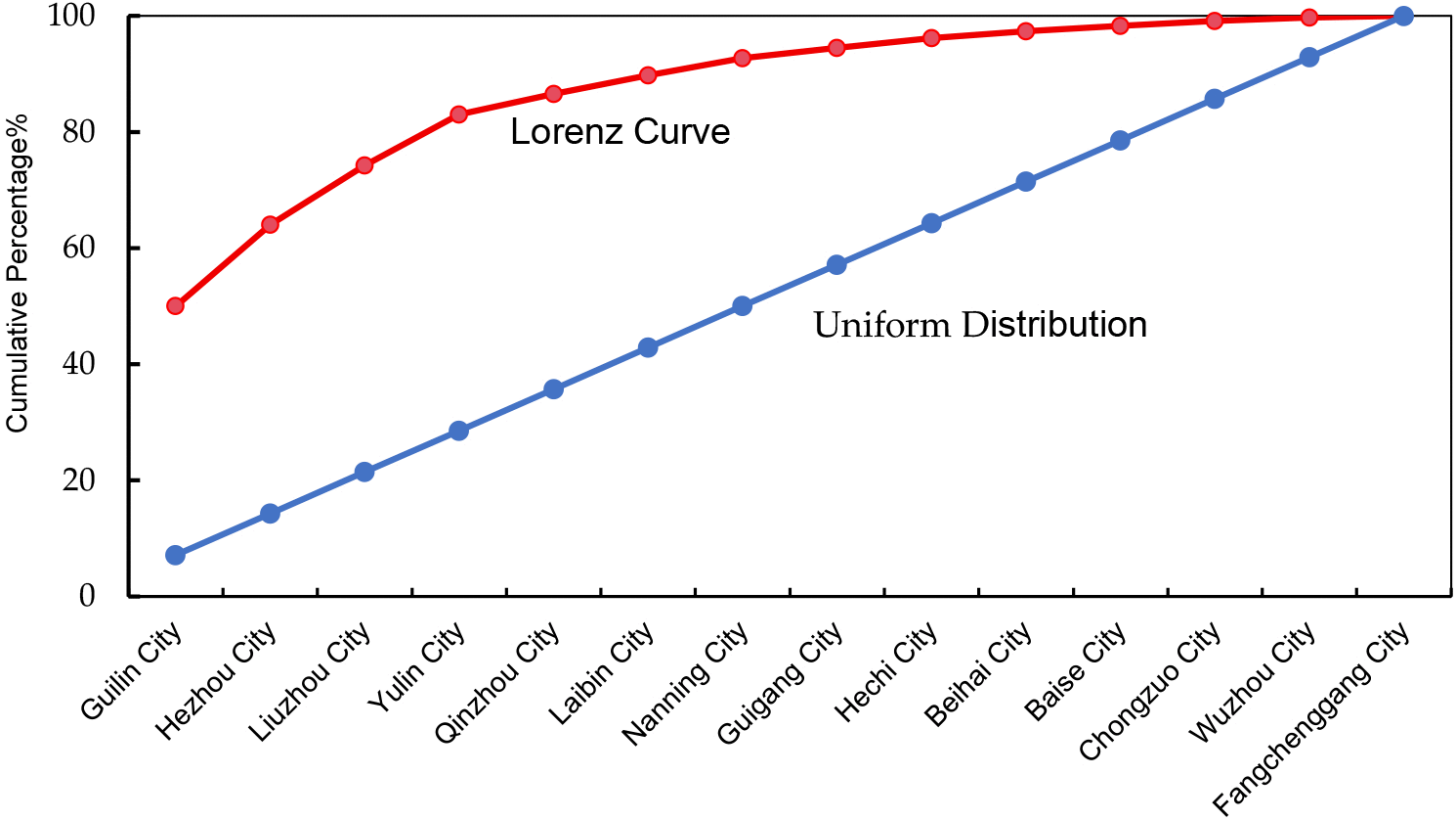
Lorenz curve of the spatial distribution.

*S* = 0.731. *S* reflects the degree of distributional equity across various cities within the study area. The results indicate a notable unevenness in their spatial allocation. The Lorenz curve (Fig 1) displays a marked deviation from the line of perfect equality, characterized by a pronounced curvature, further confirming the spatial imbalance among the sub-regions. Specifically, the combined proportion in the four prefecture-level cities of Guilin, Hezhou, Liuzhou, and Yulin accounts for as high as 83.04%. In contrast, cities such as Chongzuo, Wuzhou, Fangchenggang, and Beihai contain relatively few traditional villages.

### 3.3. Spatial distribution density

**Fig 2:**
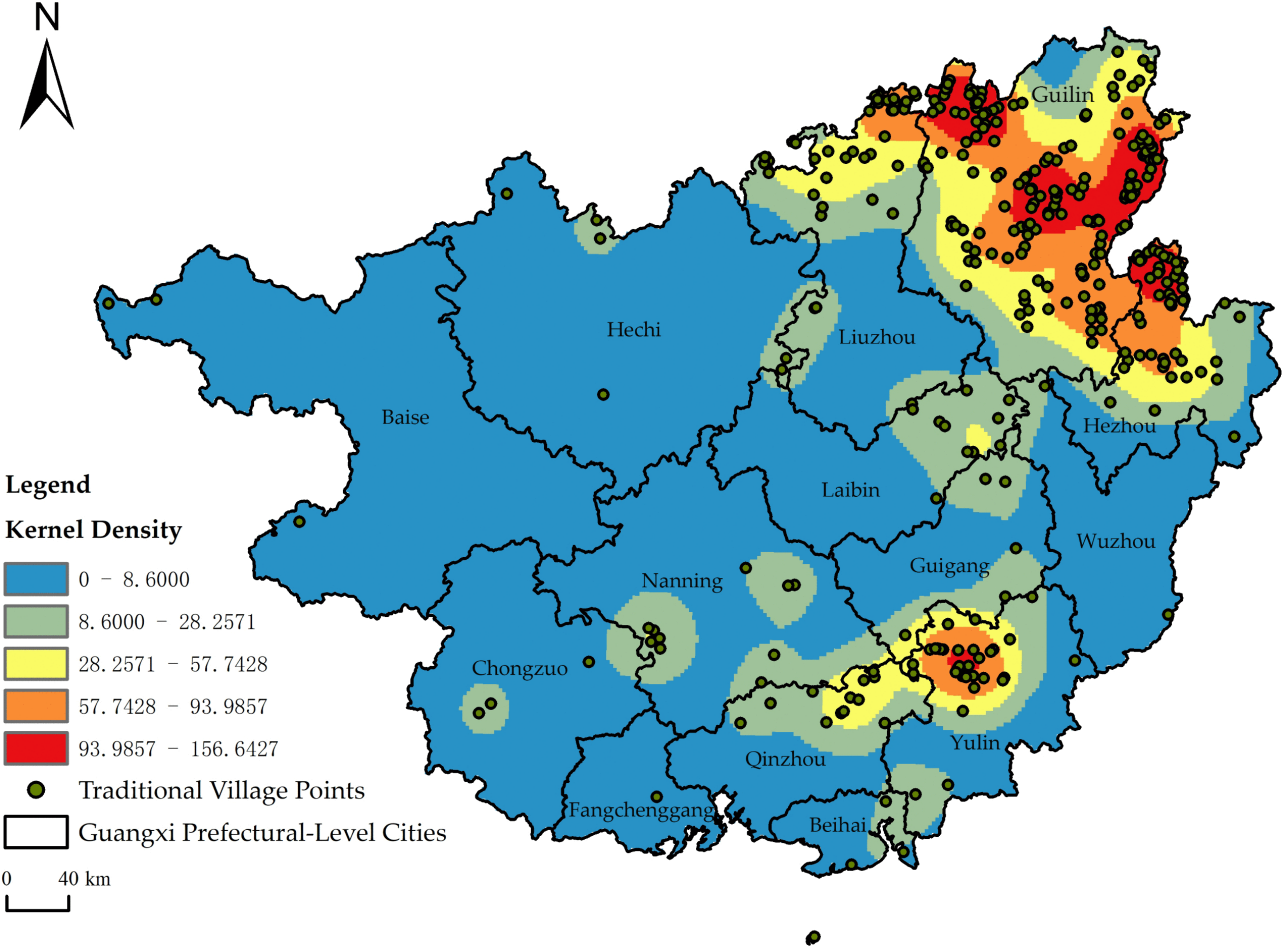
Kernel density distribution (Source of base map: reprinted from Tiandi Map).

KDE was performed using ArcGIS 10.8 software (Fig 2). The analysis reveals a pronounced concentration, predominantly clustered in the four cities of Guilin, Liuzhou, Hezhou, and Yulin. The spatial distribution exhibits a “one-primary core, three-secondary cores and dual spatial belts” pattern. That is, with Guilin as the main core, Hezhou, Liuzhou and Yulin as the secondary cores, and the distribution belts of Guilin–Liuzhou–Hezhou and Yulin–Qinzhou. Guilin, underpinned by the natural foundation of the karst landforms of northern Guangxi and the Lijiang River basin and further enriched by the cultural lineage of a historic and cultural city, has a large number of concentrated well-preserved ethnic minority villages and clusters of traditional dwellings. Liuzhou, leveraging its advantages as a transportation hub in central Guangxi and the long-standing settlement traditions of ethnic groups such as the Zhuang and Dong, has formed a village agglomeration core characterized by multi-ethnic cultural integration. As a cultural transition zone at the border of Hunan, Guangdong, and Guangxi, Hezhou has retained traditional villages reflecting the convergence of Hakka culture and Baiyue culture. Yulin, by contrast, has developed concentrated village clusters supported by the agricultural base of the plains in southeastern Guangxi and the distinctive features of its qiaoxiang (overseas Chinese hometown) culture.

## 4. Analysis of driving factors based on OPGD

### 4.1 Construction of the indicator system

The emergence and evolution of villages are shaped by the interplay of natural conditions, economic dynamics, and social factors. Initial village siting is predominantly determined by natural dimensions. Subsequent village evolution and development are driven by economic and social dimensions. The economic dimension facilitates village expansion and functional transformation, while the social dimension reshapes spatial vitality within villages. These interrelated and interacting factors collectively shape the spatial distribution pattern.

**Fig 3:**
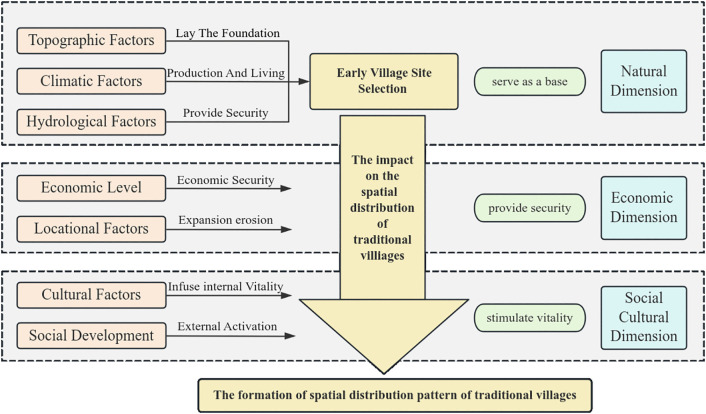
Conceptual framework for the formation of the village spatial distribution pattern.

The spatial distribution pattern results from synergistic interactions among physical geography, historical–cultural contexts, and socio-economic factors, with the logical framework of village evolution illustrated in Fig 3. Building upon this framework and prior scholarship [6,10,20,25,41,42], this study establishes a multidimensional indicator system incorporating Guangxi’s distinct ethnic-regional characteristics, structured across natural, economic, and socio-cultural dimensions through 7 primary and 12 secondary indicators (Table 2). Based on the ordinary least squares (OLS) regression diagnostics, explanatory variables were screened using multicollinearity criteria (VIF < 7.5, equivalently tolerance > 0.1) to mitigate estimation instability arising from collinearity among variables. Crucially, this system integrates understudied ethnic–cultural factors—including ethnic minority population ratios and intangible cultural heritage abundance—which demonstrate significant indicative roles in characterizing the formation, transformation, and development trajectories in ethnic minority regions. Explanatory descriptions of the 12 secondary indicators are provided in Table 3.

**Table 2:** Construction of an indicator system for factors influencing the spatial distribution of traditional villages and multicollinearity diagnostics.

Dimension	Influencing factors	Influence indicator	Vif	Tolerance
**Natural dimension**	Topographic	Elevation X_1_	3.4473	0.2901
		Slope gradient X_2_	1.5191	0.6583
		Slope aspect X_3_	1.0033	0.9967
	Climatic	Mean annual temperature X_4_	2.4898	0.4016
		Mean annual precipitation X_5_	1.9763	0.506
	Hydrological	River network density X_6_	1.1373	0.8793
**Economic dimension**	Economic	Mean GDP X_7_	7.1032	0.1408
		Road network density X_8_	1.1776	0.8492
	Locational	Distance to county administrative center X_9_	1.5401	0.6493
**Socio-cultural dimension**	Social development	Urbanization rate of permanent residents X_10_	3.8729	0.2582
	Cultural	Proportion of ethnic minority population X_11_	2.6609	0.3758
		Number of intangible cultural heritage items X_12_	4.6154	0.2167

**Table 3:** Description of influencing factors.

influencing factors	Indicator description
X_1_	The altitude of the region reflects the degree of topographic undulation
X_2_	The degree of land surface inclination reflects the difficulty of land utilization and construction
X_3_	Reflect the distribution of light and vegetation
X_4_	Regional heat conditions
X_5_	The degree of humidity in the area
X_6_	Indicates the abundance of water resources
X_7_	Reflect the economic level of the region
X_8_	It represents the convenience of transportation
X_9_	The straight-line distance between a village and the administrative center of a county reflects the situation of resource acquisition and policy radiation
X_10_	The proportion of urban population reflects the level of urbanization
X_11_	Reflect the national culture and cultural diversity
X_12_	Reflect the cultural level and the intensity of cultural inheritance and protection

### 4.2 Driving factor analysis

#### 4.2.1 Natural dimension.

**Fig 4:**
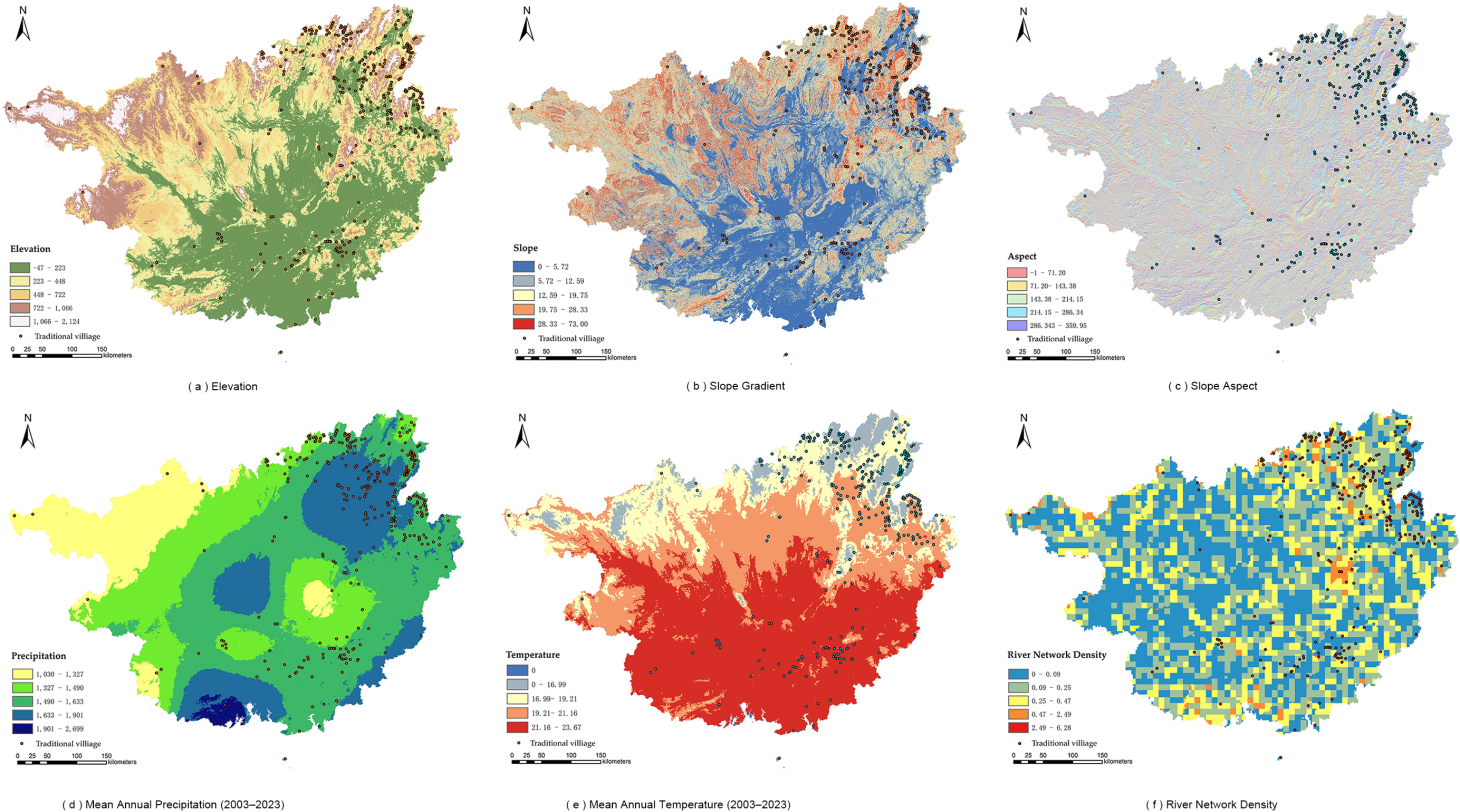
Relationship between natural dimension and spatial distribution of traditional villages (Source of base map: reprinted from Tiandi Map).

The natural environment constitutes the fundamental condition influencing human settlement, production and daily life, as well as the formation and development of villages. The results of the ArcGIS overlay analysis are shown in Fig 4.

Fig 4(a) and Fig 4(b) exhibit similar trends, indicating that traditional villages in Guangxi are primarily distributed in low- to mid-level areas with elevations below 722 m and slopes less than 19.75°, with a smaller proportion located in the high-altitude and steep-slope areas of northeastern Guangxi. Fig 4(c) shows that villages are mainly distributed in areas with aspects greater than 214.15°, which largely corresponds to sunny slopes. Fig 4(d) demonstrates that villages are concentrated in regions with precipitation ranging from 1321 to 1907 mm, where water resources are abundant. Fig 4(e) reveals that villages are situated in areas with mean annual temperatures between 16.99°C and 23.67°C, characterized by a warm and pleasant climate. Fig 4(f) illustrates that villages are distributed in regions with water network density ranging from 0.25 to 2.47, showing a pronounced tendency to be located near water. In summary, the distribution of traditional villages in Guangxi is influenced by topographic factors such as elevation and slope, while also being closely associated with climate (temperature and humidity conditions) and hydrological resources. Overall, it exhibits a spatial pattern characterized as “gentle terrain, proximity to water, and preference for warmer conditions.”

#### 4.2.2 Economic dimension.

**Fig 5:**
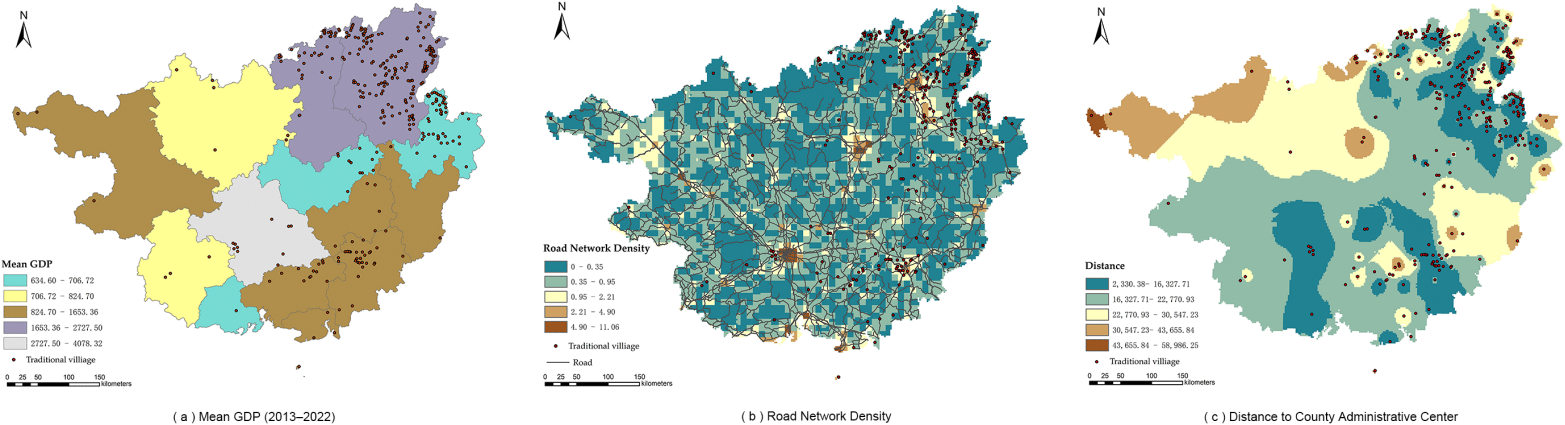
Relationship between the economic dimension and the distribution of traditional villages (Source of base map: reprinted from Tiandi Map).

The level of economic development is positively associated with the preservation of traditional villages. As shown in Fig 5(a), traditional villages in Guangxi are mainly distributed in regions with an average annual GDP above CNY 165.336 billion. This distribution is closely related to a stronger awareness of cultural heritage protection and greater policy support in economically developed regions, rather than passive preservation in economically underdeveloped regions due to delayed development. Fig 5(b) shows that villages are predominantly distributed in areas with low to medium levels of road network density, yet they are generally aligned along road networks, ensuring convenient transportation. Fig 5(c) illustrates that villages are significantly concentrated near county-level administrative centers, where they are strongly influenced by policy incentives and human intervention in preservation.

In summary, within the Guangxi region, traditional villages are mainly distributed in areas with relatively higher levels of economic development, aligned along transportation networks, and located near county-level administrative centers. Policy support and financial investment play a crucial role in the preservation of traditional villages.

#### 4.2.3 Socio-cultural dimension.

**Fig 6:**
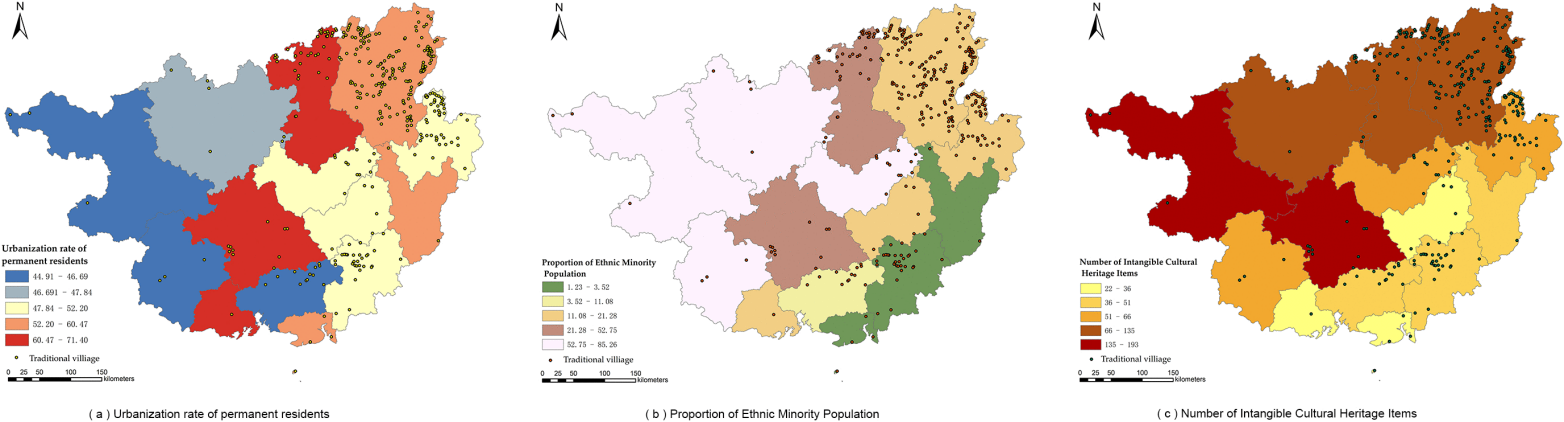
Relationship between the socio-cultural dimension and the distribution of traditional villages (Source of base map: reprinted from Tiandi Map).

The socio-cultural dimension is closely related to the distribution of traditional villages. As shown in Fig 6(a), traditional villages in Guangxi are mainly distributed in areas with relatively high levels of urbanization,i.e., where the urbanization rate exceeds 47.84%. Fig 6(b) shows that regions with an ethnic minority population proportion greater than 11.08% exhibit densely distributed villages, suggesting that cultural identity among ethnic minorities plays a positive role in the preservation of traditional architecture. Fig 6(c) reveals that villages are significantly distributed in areas with a greater abundance of intangible cultural heritage, indicating that support from cultural policies can effectively promote the preservation and sustainable utilization of traditional villages.

In summary, within Guangxi, regions characterized by higher urbanization rates, a higher proportion of ethnic minority populations, and rich intangible cultural heritage demonstrate better preservation of traditional villages and thereby retain a greater number of villages.

### 4.3. Parameter optimization

The choice of spatial scale directly affects the effectiveness of driver analysis. An overly large scale may dilute critical spatial details, thereby homogenizing the influence patterns of dominant factors, whereas an overly small scale is more susceptible to local heterogeneity, leading to confounded and unstable results. Drawing upon existing scholarly research and the contextual characteristics of the study area [43–44], five spatial grid scales were selected: 3 km × 3 km, 5 km × 5 km, 8 km × 8 km, 10 km × 10 km, and 12 km × 12 km. The variation in influencing factor strength was analyzed by comparing the magnitudes of the q-values at the 90th percentile. Results are presented in Table 4. The analysis reveals that the q-value reached its peak of 0.9366 under the 8 km × 8 km grid scale, indicating this as the optimal spatial scale for obtaining higher analytical precision.

**Table 4:** Comparative analysis of q-values at the 90th percentile across spatial scales.

Influence indicator	3km	5km	8km	10km	12km
**X**_**1**_	**0.0309**	**0.0306**	0.0286	**0.0342**	**0.0366**
**X**_**2**_	**0.0090**	**0.0093**	0.0076	**0.0175**	**0.0119**
**X**_**3**_	**0.0010**	**0.0009**	0.0029	**0.0068**	**0.0045**
**X**_**4**_	**0.2146**	**0.2162**	0.2241	**0.2138**	**0.2175**
**X**_**5**_	**0.1916**	**0.1899**	0.1969	**0.1953**	**0.1949**
**X**_**6**_	**0.0288**	**0.0303**	0.0282	**0.0305**	**0.0285**
**X**_**7**_	**0.9839**	**0.9840**	0.9839	**0.9839**	**0.9846**
**X**_**8**_	**0.0085**	**0.0072**	0.0097	**0.0071**	**0.0179**
**X**_**9**_	**0.0985**	**0.0949**	0.0978	**0.1051**	**0.0975**
**X**_**10**_	**0.6568**	**0.6599**	0.6587	**0.6614**	**0.6552**
**X**_**11**_	**0.6483**	**0.6502**	0.6537	**0.6514**	**0.6469**
**X**_**12**_	**0.9664**	**0.9672**	0.9675	**0.9668**	**0.9673**
**q-value at the 90th percentile**	**0.9355**	**0.9364**	0.9366	**0.9362**	**0.9361**

**Fig 7:**
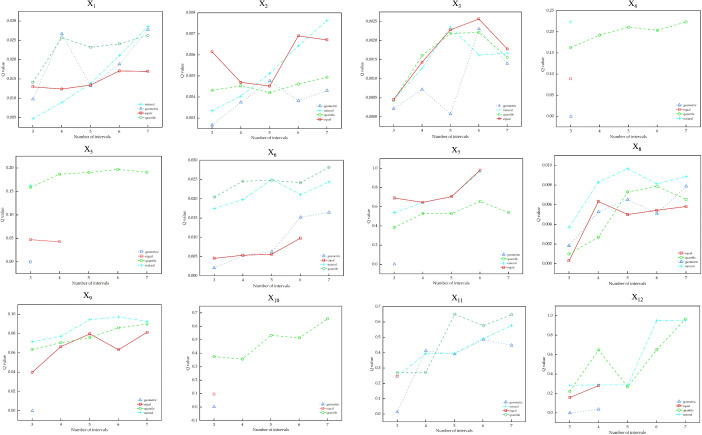
Discretization methods and interval quantities.

Given the spatial extent of the study area and the sample size, to avoid insufficient comparison arising from too few classes and overfitting caused by too many classes, each continuous explanatory factor was discretized into 3–7 classes using *RStudio* [45–46]. Because the data are unevenly distributed, discretization based on the standard deviation method produced invalid results; therefore, four discretization methods were adopted: equal interval, natural breaks, quantile, and geometric interval. The optimal discretization scheme (method and number of classes) for each continuous influencing factor was determined by maximizing its explanatory power, thereby finalizing the parameter configuration for the subsequent analysis. For example, river network density (X₆) was discretized into seven classes using the quantile method (Fig 7), whereas road network density (X₈) was discretized into five classes using the natural breaks method (Fig 7). The statistical results for the optimal parameter combinations of all driving factors are reported in Table 5.

**Table 5:** The specific classification intervals and the number of classification samples.

Driving factors	Discrete methods	The number of partitions	Unit	Classification interval	Number of samples in the interval	proportion （%）
X_1_	Natural breaks classification	7	m	[0,173] (173,331] (331,509] (509,714] (714,937] (937,1210] (1210,1780]	1184 862 567 469 338 202 78	32.00 23.30 15.32 12.68 9.14 5.46 2.11
X_2_	Natural breaks classification	7	Degree(°)	[0,4.06] (4.06,8.72] (8.72,13.6] (13.6,18.8] (18.8,24.7] (24.7,32.2] (32.2,51.3]	1003 656 604 571 459 306 101	27.11 17.73 16.32 15.43 12.41 8.27 2.73
X_3_	Equal interval classification	6	Degree(°)	[-1,59.1] (59.1,119] (119,179] (179,239] (239,300] (300,360]	629 571 651 662 545 642	17.00 15.43 17.59 17.89 14.73 17.35
X_4_	Quantile classification	7	Celsius（℃）	[0,18.1] (18.1,19.3] (19.3,20.4] (20.4,21.3] (21.3,22] (22,22.4] (22.4,23.5]	529 528 529 528 529 528 529	14.30 14.27 14.30 14.27 14.30 14.27 14.30
X_5_	Quantile classification	6	mm	[0,1340] (1340,1460] (1460,1530] (1530,1590] (1590,1660] (1660,2160]	619 616 625 614 613 613	16.73 16.65 16.89 16.59 16.57 16.57
X_6_	Quantile classification	7	km/km²	[0,0.0255] (0.0255,0.0794] (0.0794,0.14] (0.14,0.195] (0.195,0.243] (0.243,0.325] (0.325,1.34]	925 466 459 463 463 461 463	25.00 12.59 12.41 12.51 12.51 12.46 12.51
X_7_	Equal interval classification	6	100 million yuan	(0,680] (680,1360] (1360,2040] (2040,2720] (2720,3400] (3400,4080]	414 2025 198 430 286 347	11.19 54.73 5.35 11.62 7.73 9.38
X_8_	Natural breaks classification	5	km/km²	[0,0.515] (0.515,1.47] (1.47,3.45] (3.45,7.1] (7.1,11.1]	2444 1105 114 32 5	66.05 29.86 3.08 0.86 0.14
X_9_	Natural breaks classification	6	m	[0,11700] (11700,17500] (17500,22300] (22300,27600] (27600,34800] (34800,58700]	166 878 1282 716 506 152	4.49 23.73 34.65 19.35 13.68 4.11
X_10_	Quantile classification	7	%	[0,46.7] (46.7,47.8] (47.8,50.2] (50.2,52.1] (52.1,55.1] (55.1,71.2] (71.2,71.4]	752 797 212 381 596 615 347	20.32 21.54 5.73 10.30 16.11 16.62 9.38
X_11_	Quantile classification	5	%	[0,16.1] (16.1,21.3] (21.3,76.7] (76.7,83.9] (83.9,85.3]	789 701 845 1093 272	21.32 18.95 22.84 29.54 7.35
X_12_	Natural breaks classification	7	--	[0,48] (48,57] (57,118] (118,123] (123,135] (135,173] (173,193]	685 464 920 286 430 568 347	18.51 12.54 24.86 7.73 11.62 15.35 9.38

### 4.4. Single factor detection analysis

Factor detection was performed using OPGD to identify the contribution degree (q-value) and statistical significance (p < 0.05) of drivers influencing in the study area (Table 6). According to the detection results, aspect failed to pass the significance test and was therefore not representative, whereas the remaining 11 factors exhibited significant differences in their explanatory power for the spatial distribution of villages. Based on the magnitude of the q statistic, these factors can be classified into three levels: strong, moderate, and weak.

**Table 6:** OPGD single-factor detection results.

Influence indicator	q-values	p-values
**X**_**1**_	0.0286^**^	0.0000
**X**_**2**_	0.0076^**^	0.0001
**X**_**3**_	0.0029	0.0625
**X**_**4**_	0.2241^**^	0.0000
**X**_**5**_	0.1969^**^	0.0000
**X**_**6**_	0.0282^**^	0.0000
**X**_**7**_	0.9839^**^	0.0000
**X**_**8**_	0.0097^**^	0.0000
**X**_**9**_	0.0978^**^	0.0000
**X**_**10**_	0.6587^**^	0.0000
**X**_**11**_	0.6537^**^	0.0000
**X**_**12**_	0.9675^**^	0.0000

Note: ** denotes statistical significance at the 0.001 level; * indicates statistical significance at the 0.05 level.

The strong factors (q > 0.6) include average GDP (X₇), the number of intangible cultural heritage items (X₁₂), the urbanization rate of permanent residents (X₁₀), and the proportion of ethnic minority population (X₁₁), with q values of 0.9839, 0.9675, 0.6587, and 0.6537, respectively. All four factors passed the highly significant test (p < 0.001) and constitute the core drivers of spatial differentiation in villages. The high q value of average annual GDP (X₇) indicates that the level of regional economic development provides decisive support for the persistence and conservation of ancient/traditional villages; economically stronger regions are better able to undertake village landscape restoration and cultural inheritance initiatives. The high q value for the number of intangible cultural heritage items (X₁₂) suggests that, as key carriers of ethnic minority culture and traditional craftsmanship, the spatial distribution of villages closely aligns with the agglomeration pattern of intangible cultural heritage resources, and that the demand for cultural transmission is a crucial internal driver of village location and continuity. The high q values for the urbanization rate of permanent residents (X₁₀) and the proportion of ethnic minority population (X₁₁) further confirm that the distribution of traditional villages is associated both with spatial differences in the regional urbanization process and with the cultural settlement characteristics of ethnic minority communities, thereby forming a core driving pattern of “cultural agglomeration + economic support.”

The moderate factors (0.1 < q < 0.6) include mean annual temperature (X₄) and mean annual precipitation (X₅), with q values of 0.2241 and 0.1969, respectively; both pass the highly significant test (p < 0.001). The detection results for these two climatic factors indicate that village distribution shows a certain degree of adaptation to regional hydrothermal conditions. A warm and humid climatic environment provides a suitable natural basis for village construction and long-term persistence, which is consistent with the siting logic of traditional villages in southern China that aligns with favorable climatic conditions.

The weak factors (q < 0.1) include elevation (X₁), slope (X₂), aspect (X₃), river network density (X₆), road network density (X₈), and distance to the county-level administrative center (X₉). All of these factors have q values below 0.1, indicating that their individual explanatory power for village distribution is limited.

### 4.5. Factor interaction analysis

**Fig 8:**
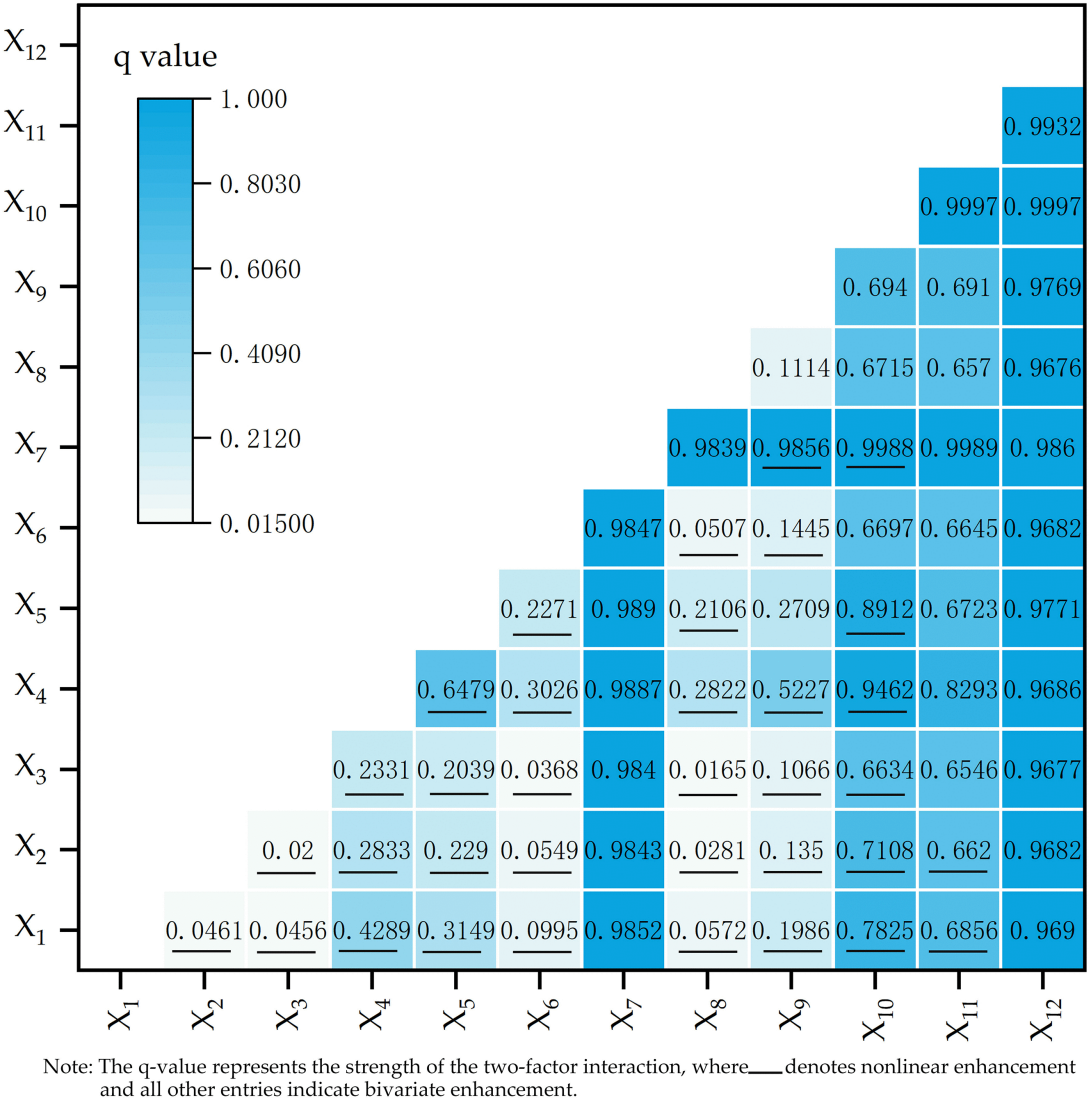
Results of factor interaction detection.

OPGD interaction detection evaluates the existence, strength, direction (enhancing/weakening), and functional nature (linear/nonlinear) of pairwise factor interdependencies. Bivariate synergistic enhancement is confirmed when the interaction term satisfies q (X_1,_ X_2_)>Max(q(X_1_), P(X_2_)), indicating that the combined effect surpasses the influence of individual factors,while nonlinear enhancement emerges under the condition q(X_1_∩X_2_)>q(X_1_)+q(X_2_), revealing a hierarchical progression in efficacy in the order nonlinear enhancement > bivariate enhancement > isolated factor effects. As shown in Fig 8, the interactions among factors exhibit varying degrees of nonlinear enhancement and two-factor enhancement. This indicates that the spatial distribution pattern of traditional villages in the study area results from the joint effects of multiple factors, and the combined effect of any two factors is significantly stronger than the independent effect of a single factor.

Nonlinear enhancement combinations exhibit a strong synergistic amplification effect between core factors and auxiliary factors. Such combinations are predominantly observed in pairings between strongly influential core factors and other factors, and they display the highest interaction intensity, constituting key compound factor pairs that drive spatial differentiation in village distribution. For example, for X₁ (elevation) ∩ X₁₀ (urbanization rate of permanent residents), the q value of the single factor X₁ is only 0.0286, whereas that of X₁₀ is 0.6587; the sum of the two single-factor q values is 0.6873, while the interaction q increases to 0.7825. This value markedly exceeds the sum of the single-factor q values, indicating an amplification effect whereby a core socio-economic factor magnifies the influence of a natural auxiliary factor. In general, weak factors from different dimensions can, when coupled with core factors, generate effective driving forces for village distribution through nonlinear superposition.

Two-factor enhancement combinations exhibit a dominant pattern driven by core factor ∩ core factor interactions under broad-based synergy, covering the vast majority of factor pairings. Although these combinations do not reach the intensity of nonlinear enhancement, they still achieve a marked increase over single-factor effects through synergistic interactions. Notably, pairings between strongly influential core factors show the highest interaction intensity. For instance, the interaction q value for X₇ (average annual GDP) ∩ X₁₂ (number of intangible cultural heritage items) reaches 0.9860, and that for X₁₀ (urbanization rate of permanent residents) ∩ X₁₁ (proportion of ethnic minority population) is as high as 0.9997; both exceed the corresponding single-factor q values. These results demonstrate that synergy between economic and cultural factors, as well as between social and ethnic factors, constitutes a strong compound driving force shaping village distribution. Such synergistic effects further reinforce the central roles of economic support, cultural inheritance, and social development in sustaining villages.

Significant synergistic effects were observed between economic development, cultural factors, the natural environment, and social development. This finding demonstrates that the development of traditional villages is typically accompanied by cultural accumulation. Regions proximate to county administrative centers and exhibiting robust economic development possess greater potential for preserving or fostering traditional villages. Concurrently, the analysis reveals that water systems and favorable climatic conditions collaboratively shape habitable environments, consequently shaping the spatial distribution and locational choices of villages. Notably, both single-factor and interaction analyses consistently identify economic and cultural factors as the dominant influences shaping spatial distribution. Their impact significantly outweighs that of other dimensions.

## 5. Mechanisms influencing the spatial distribution of traditional villages in Guangxi

**Fig 9:**
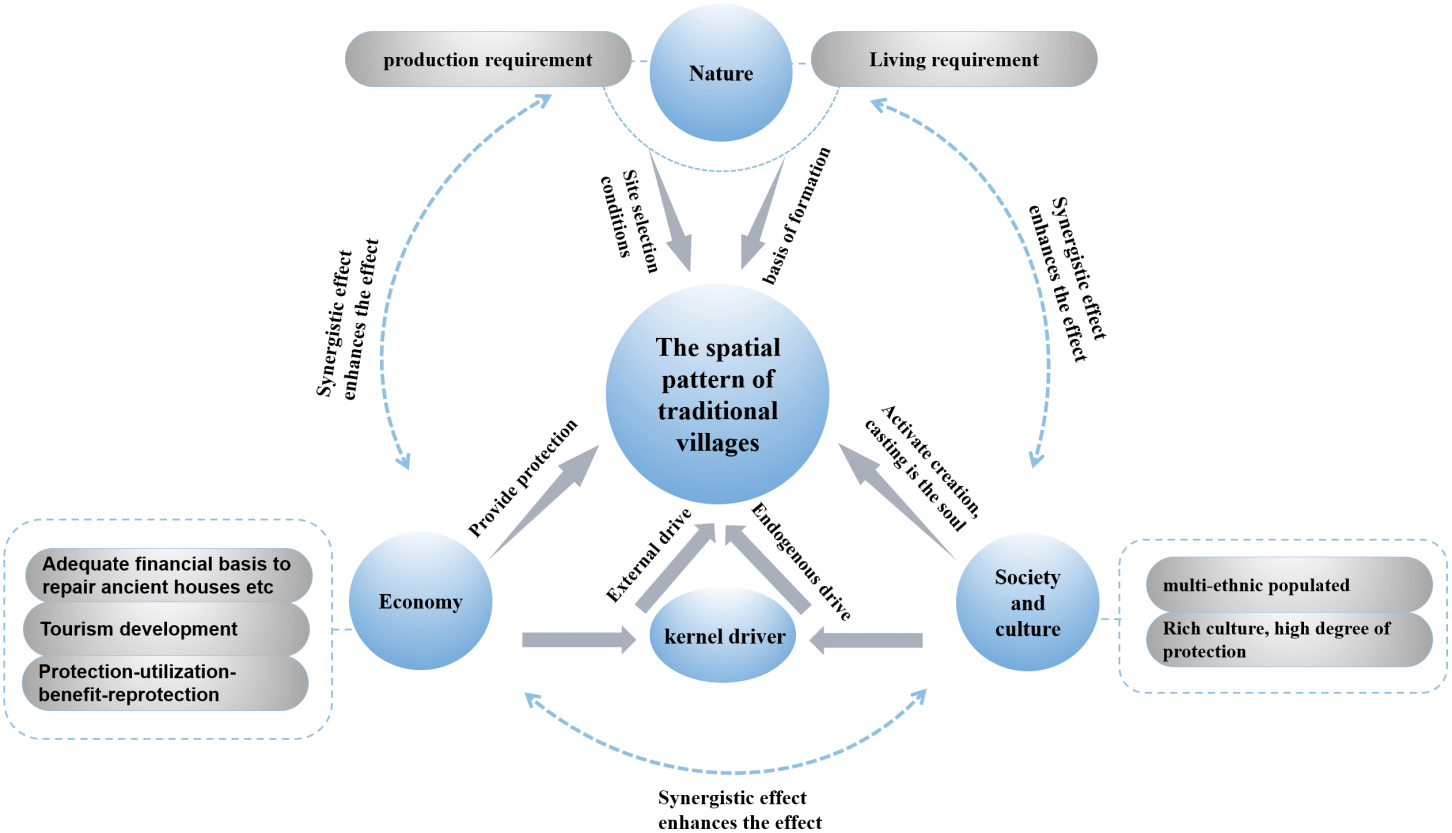
Mechanism of influencing factors on the spatial pattern of traditional villages in Guangxi.

The spatial distribution of traditional villages in Guangxi results from the interactions among natural conditions, economic development, and socio-cultural factors, exhibiting an overall pattern characterized by the dominance of core factors and cross-dimensional synergistic enhancement (Fig 9).

### 5.1. Natural conditions as the foundational basis

The natural geographic environment provides the fundamental basis for the initial siting and formation of villages. Guangxi’s warm and humid subtropical monsoon climate supplies abundant precipitation and heat, meeting the requirements of agricultural production and securing the material foundations necessary for village existence and development. Meanwhile, natural conditions serve as key criteria for village siting. The widespread karst landforms in Guangxi have shaped traditional villages with distinctive regional characteristics, reflecting the wisdom of earlier generations in selecting settlement locations and giving rise to unique vernacular settlement landscapes.

### 5.2. Economic strength as a supporting guarantee

Economic development plays a safeguarding role in the transformation and conservation of traditional villages and constitutes a key external driving force. For example, a large number of ancient/traditional villages are concentrated around cities such as Guilin and Liuzhou, where the average annual GDP is relatively high. These areas have sufficient fiscal resources to support the restoration of traditional dwellings, village landscape improvement, and the construction of cultural heritage conservation facilities. In addition, strong economic capacity underpins tourism development and the commercialization of intangible cultural heritage, thereby enabling a virtuous cycle of “conservation–utilization–revenue–reconservation.” By contrast, economically less developed areas such as Chongzuo and Fangchenggang, constrained by limited conservation funding and development capacity, host relatively few traditional villages and find it difficult to achieve large-scale agglomeration.

### 5.3. Socio-cultural factors as the animating core

Socio-cultural elements play a role in activating and shaping the distribution pattern of traditional villages during their development. As an internal core driving force, they endow traditional villages with enduring cultural meaning and identity. In Guangxi, abundant intangible cultural heritage resources among ethnic minority communities—including traditional craftsmanship, folk festivals, and everyday customs—are deeply embedded in village spatial organization, architectural styles, and social structures. For instance, Dong drum towers and Zhuang stilted (ganlan-type) houses are not only residential spaces but also important venues for cultural transmission. The agglomeration of intangible cultural heritage encourages continued settlement, strengthens cultural centripetal forces, and promotes village growth, making areas such as Hezhou and Yulin both intangible cultural heritage-rich zones and secondary cores of village concentration. By contrast, in areas where cultural transmission has been disrupted or where intangible cultural heritage is scarce, villages may lose their distinctive internal cultural cohesion, become less resilient to the pressures of modernization, and are more likely to decline or even disappear. In addition, ethnic minority groups in Guangxi—such as the Zhuang, Dong, and Yao—often exhibit strong ethnic identity and a preference for clustered settlement. As a result, village layouts frequently concentrate around culturally significant sites. Examples include stilt-house clusters in Miao villages and the spatial organization of Dong stockaded villages, which not only adapt to local environmental conditions but also reinforce group cohesion. Many of these ethnic minority settlement areas have retained relatively intact traditional modes of production and daily life and have been less affected by modernization, thereby constituting the primary concentration zones of traditional villages. Conversely, in places with a higher degree of ethnic integration and where traditional group structures have been disrupted, ethnic cultural characteristics of villages have gradually weakened, accompanied by a corresponding decline in the number of villages preserved.

## 6. Protection strategies for traditional villages

### 6.1. Spatially differentiated conservation strategies

Given the pronounced concentration of villages in Guilin (the primary core) and in Liuzhou, Hezhou, and Yulin (secondary cores), resources should be prioritized toward these core areas while also safeguarding the cultural integrity of peripheral regions.

(1) Primary and secondary core areas: advancing systematic and coordinated conservation.

In Guilin, building on the karst landforms, the ecological baseline of the Lijiang River basin, and its advantages as a nationally recognized historic and cultural city, conservation efforts should prioritize the architectural character of ethnic minority villages and their landscape–settlement configuration. Development intensity in surrounding areas should be strictly controlled to curb the encroachment of disorderly urban expansion on village space. In Liuzhou, Hezhou, and Yulin, conservation should highlight multi-ethnic cultural integration by consolidating resources such as the Miao stilt-house architecture in Rongshui, the Dong drum tower–wind-and-rain bridge system in Sanjiang, and Zhuang ganlan-type dwellings in Liucheng. These assets can be leveraged to develop ethnic cultural display corridors and to form cultural clusters that integrate living heritage transmission, study-tour experiences, and tourism-related consumption.

(2) Distribution corridors: strengthening linear linkage and resource integration.

Along the Guilin–Liuzhou–Hezhou corridor and the Yulin–Qinzhou–Nanning corridor, efforts should be made to identify cultural linkages among villages and to enhance spatial connectivity along the routes. A corridor-based conservation network should be established to avoid cultural discontinuities and landscape fragmentation that may result from isolated conservation initiatives.

(3) Peripheral and vulnerable areas: implementing targeted, gap-filling support.

In areas such as Chongzuo, Wuzhou, Fangchenggang, and Beihai—where the number of villages is small but cultural distinctiveness is pronounced—a registration and documentation program for scattered villages should be carried out. Priority should be given to emergency (salvage) conservation of distinctive architectural typologies and intangible cultural heritage remnants. Meanwhile, successful practices from core areas should be introduced in a context-sensitive manner to cultivate small-scale cultural nodes with strong local identity, thereby preventing conservation blind spots in which areas with fewer villages are overlooked.

### 6.2. Strategies for strengthening core driving forces

The sustainable continuity of traditional villages depends on the dual activation of economic support capacity and endogenous cultural vitality.

(1) Establishing a virtuous cycle of “conservation–utilization–revenue.”

Fiscal support should be further tilted toward core areas, with priority given to the repair of traditional dwellings, village landscape improvement, and upgrades to heritage-related facilities. Economically stronger cities such as Guilin and Liuzhou should be encouraged to support less-developed areas through financial assistance, technology transfer, and personnel training. Innovative models integrating culture and tourism can be promoted to transform intangible cultural heritage (ICH) resources into product systems that are experienceable, marketable, and communicable. Examples include immersive theatrical experiences based on the Dong Grand Song, Zhuang brocade and embroidery workshops, and Yao medicinal-bath wellness programs. These initiatives can help achieve “conservation through use” and “heritage safeguarding supported by cultural industries.”

(2) Improving diversified investment and long-term safeguard mechanisms.

Social capital should be guided to participate in an orderly manner. A Guangxi Traditional Village Conservation and Development Fund can be established, with exploration of hybrid operational models that combine public-interest objectives with market mechanisms (“public welfare + market”). Investment–return and risk-sharing arrangements should be improved to ensure the sustainability of conservation funding.

(3) Safeguarding the roots of ethnic culture.

In ethnic minority settlement areas, conservation should prioritize traditional modes of production and daily life, systems of festival and ritual practices, and settlement spatial structures. In parallel, village chronicles should be compiled, ethnographic records and texts on customary practices should be organized, and digital cultural resource repositories should be developed to enable systematic documentation, long-term preservation, and innovative dissemination of cultural heritage.

### 6.3. Optimizing the natural foundation to consolidate the conservation base

Given the observed spatial regularities—namely, that villages are generally distributed at elevations below 722 m, on slopes below 19.75°, on warmer sun-facing aspects, and in proximity to water—topographic and geomorphological protection should be strengthened. Mountain cutting and valley filling should be strictly controlled, and systematic measures should be implemented to prevent and mitigate geohazards such as landslides and debris flows. For the small number of villages located in high-altitude and steep-slope areas (e.g., in northeastern Guangxi), priority should be given to upgrading basic infrastructure, including water supply, electricity, and telecommunications, to enhance resilience to natural risks and to prevent population outflow and village hollowing driven by environmental constraints.

### 6.4. Activating synergistic effects among multidimensional factors

Conservation strategies should move beyond single-dimension interventions and proactively leverage nonlinear enhancement effects among natural, economic, and socio-cultural elements, thereby activating synergies across dimensions. For example, building on favorable terrain (gentle slopes and low elevations) while incorporating infrastructure improvements and policy support associated with urbanization can enhance the overall carrying capacity of villages. Moreover, integrating warm and humid climatic conditions with the settlement traditions of ethnic minority communities can help develop ethnic villages with dual advantages in ecology and culture.

## 7. Conclusions and discussion

This study investigated 342 nationally designated traditional villages in Guangxi, employing NNI, imbalance index, KDE, and OPGD to characterize their spatial distribution and identify dominant influencing factors. The main conclusions drawn are as follows:

(1) Traditional villages display a clearly clustered spatial distribution pattern throughout the study area.
(2) Their distribution demonstrates significant unevenness, with 83.04% concentrated in four prefecture-level cities: Guilin, Hezhou, Liuzhou, and Yulin. Conversely, regions including Chongzuo, Wuzhou, Fangchenggang, and Beihai contain relatively few traditional villages.
(3) The spatial distribution shows a “one-primary core, three-secondary cores and dual spatial belts” pattern. The primary core comprises high-density clusters in Guilin. Secondary cores are in Hezhou, Liuzhou, and Yulin. Spatial belts are the Guilin–Liuzhou–Hezhou development belt and the Yulin–Qinzhou development belt.
(4) The spatial distribution of traditional villages in the study area is jointly shaped by three forces: natural conditions as the foundational basis, economic strength as the supporting guarantee, and socio-cultural factors as the animating core. The coordination and mutual reinforcement among these elements promote the distinctive development and continuity of traditional villages.
(5) Accordingly, this study proposes an integrated four-component conservation framework oriented toward spatially differentiated protection, reinforcement of core driving forces, optimization of the natural foundation, and activation of multidimensional synergy.

From a macro-level perspective, this study quantitatively examines the spatial pattern of traditional villages in ethnic minority regions, identifies the major driving factors shaping this pattern and their interaction effects, and elucidates the mechanisms influencing the spatial distribution of traditional villages. On this basis, corresponding conservation strategies are proposed. It excludes micro-level influences such as intra-village structures and residents’ educational attainment and lacks in-depth investigation into context-specific interaction mechanisms. Future research should prioritize micro-scale attributes—including morphological features, cultural significance, and heritage values—while integrating exemplar case studies to dissect the interplay of influencing factors, thereby advancing more nuanced theoretical frameworks on traditional village spatial configurations.

## Supporting information

S1 Data The first to sixth batches of Guangxi traditional village list are summarized, including its administrative region, location coordinates, and social and economic data of prefecture-level cities in Guangxi. (ZIP)

S2 Data Regional natural and social data, including elevation, temperature, precipitation, water system, road, etc. (The data set contains sub-files which are shp or tif files, which need to be opened in ArcGIS, QGIS, Gispro and other software). (ZIP)
